# Molecular characterization of HIV-1 Nef and ACOT8 interaction: insights from *in silico* structural predictions and *in vitro* functional assays

**DOI:** 10.1038/srep22319

**Published:** 2016-03-01

**Authors:** Michela Serena, Alejandro Giorgetti, Mirko Busato, Francesca Gasparini, Erica Diani, Maria Grazia Romanelli, Donato Zipeto

**Affiliations:** 1 Department of Neurosciences, Biomedicine and Movement Sciences, University of Verona, Strada le Grazie 8, 37134 Verona, Italy; 2 Department of Biotechnology, University of Verona, Strada le Grazie 15, 37134 Verona, Italy

## Abstract

HIV-1 Nef interacts with several cellular proteins, among which the human peroxisomal thioesterase 8 (ACOT8). This interaction may be involved in the endocytosis regulation of membrane proteins and might modulate lipid composition in membrane rafts. Nef regions involved in the interaction have been experimentally characterized, whereas structural details of the ACOT8 protein are unknown. The lack of structural information hampers the comprehension of the functional consequences of the complex formation during HIV-1 infection. We modelled, through *in silico* predictions, the ACOT8 structure and we observed a high charge complementarity between Nef and ACOT8 surfaces, which allowed the identification of the ACOT8 putative contact points involved in the interaction. The predictions were validated by *in vitro* assays through the development of ACOT8 deletion mutants. Coimmunoprecipitation and immunofluorescence analyses showed that ACOT8 Arg^45^-Phe^55^ and Arg^86^-Pro^93^ regions are involved in Nef association. In addition, K91S mutation abrogated the interaction with Nef, indicating that Lys^91^ plays a key role in the interaction. Finally, when associated with ACOT8, Nef may be preserved from degradation. These findings improve the comprehension of the association between HIV-1 Nef and ACOT8, helping elucidating the biological effect of their interaction.

Nef is an HIV-1 accessory protein involved in several mechanisms modulating the virus infectious cycle^1^. Some long-term non-progressor patients have been found to carry HIV-1 mutants with deletions in *nef* or with a high frequency of defective *nef* alleles^2^,^3^,^4^. Several functions of Nef have been documented in tissue cultures: Nef enhances viral infectivity and replication in PBMC^5^,^6^, alters the state of T-cell activation and macrophage signal transduction pathways^7^,^8^,^9^, inhibits the immunoglobulin class switching^10^, reduces the cell surface expression of the CD4 receptor^11^, whose internalization and degradation is essential to increase the infectivity of the released HIV-1 viral particles^12^,^13^. Finally, Nef downregulates the cell surface expression of MHC-I molecules to escape the host immune response^14^,^15^,^16^,^17^,^18^ and associates with several components of the endocytic pathways^19^.

Nef is described as a raft-associated protein, through its N-terminal myristoylation, which is necessary for its anchorage to the cell membrane^20^,^21^. Myristoylated Nef can adopt several quaternary structures as monomers, dimers and trimers and it may associate with other proteins^22^,^23^. However, myristoylation of Nef alone is insufficient for lipid binding, suggesting that more complex interactions are necessary to allow its migration and binding to the membrane^20^. An additional Nef-interacting protein is the human thioesterase 8 (ACOT8)^24^,^25^, which is a peroxisomal enzyme involved in lipid metabolism. The human *ACOT8* gene is located on chromosome 20q13.12 and codes for a 319 aa residues protein of approximately 35 kDa^24^,^26^. Due to the serine-lysine-leucine (SKL) peroxisomal targeting signal, it is localized in peroxisomes^24^,^26^,^27^. It has been shown that murine ACOT8 is inhibited by Coenzyme A (CoASH)^28^, differently from the Type-I ACOTs. Thus, the sensitivity to CoASH and the very broad substrate specificity suggest a role for this enzyme in regulating the intra-peroxisomal acyl-CoA/CoASH level in order to optimize the fatty acids flux through the β-oxidation system. In contrast to the peroxisomal Type-I ACOTs, ACOT8 shows a broad tissue expression range both in mice and humans^25^,^28^. However, the role of this enzyme in lipid metabolism is not clear. Although ACOT8 structure has not been solved by crystallography, Li and co-workers^29^ solved the three-dimensional structure of the *Escherichia coli* thioesterase II by X-ray crystallography. The latter shares about 41% of aminoacidic sequence identity with ACOT8. While *E. coli* thioesterase II is a tetramer, the human thioesterase 8 is present both in dimeric and tetrameric forms^30^. Yeast two-hybrid studies have shown that HIV-1 Nef directly interacts with ACOT8^24^,^25^. HIV-1 Nef-LAI residues from Asp^108^ to Trp^124^ (in particular Asp^108^, Leu^112^, Phe^121^, Pro^122^, Asp^123^) have been identified as essential for ACOT8 interaction^30^,^31^. It has been demonstrated that expression of ACOT8 promotes the relocalization of Nef to peroxisomes in 3T3 cells^30^. Nef/ACOT8 colocalization in peroxisomes requires the C-terminal peroxisomal targeting sequence of ACOT8. Several hypotheses were proposed to explain why HIV-1 Nef associates with ACOT8. Since it has been reported that the preferred substrates of ACOT8 are myristoyl-CoA and palmitoyl-CoA^24^, ACOT8 activity could be involved in the control of lipid modifications of proteins, which are important for their membrane anchoring and receptor internalization^31^. Previous reports showed that palmitoylation could influence the rate of endocytosis of molecules at the plasma membrane^32^,^33^,^34^. Thus, ACOT8 could act on the acylation of these proteins by regulating the intracellular level of acyl-CoA. In addition, lipid rafts are preferential sites for HIV-1 viral particles budding^35^ and it has been reported that hydrolysis of long chain fatty acyl-CoA is required for correct budding of Coat Protein Complex I (COP-I) coated vesicles^36^, whose β-COP subunit is a Nef interacting target^37^. In the absence of acyl-CoA, buds accumulate and coated vesicles fail to pinch off. Since ACOT8 hydrolyses a broad range of acyl-CoA and Nef may enhance its enzymatic activity in a dose dependent manner^25^, it is likely that the Nef-mediated ACOT8 recruitment may be involved in intracellular transport pathways modification.

So far, research studies have been focused on determining the Nef regions involved in the interaction with ACOT8. In this work, through *in silico* predictions, we modelled the human ACOT8 structure starting from the *E. coli* thioesterase to perform a docking analysis between HIV-1 Nef and ACOT8. The ACOT8 amino acids most likely involved in the interaction with Nef were predicted and several ACOT8 deletion mutants were developed and analysed for their interacting properties and ability to target Nef in the peroxisomes. Through coimmunoprecipitation and immunofluorescence assays, we observed that ACOT8 Lys^91^ is essential for Nef binding. Finally, expression analysis of Nef and ACOT8 suggests that the Nef/ACOT8 association may have a role in reducing Nef degradation.

## Results

### Computational ACOT8 model

**Figure 1:**
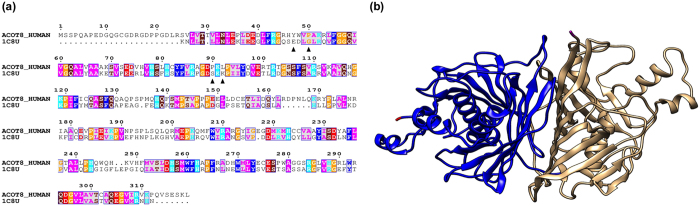
ACOT8 homology modelling. **(a)** Sequence alignment between Acyl-coenzyme A thioesterase 8 (ACOT8_HUMAN) and *E. coli* thioesterase II (1C8U). ▲ indicates aromatic and aliphatic residues (Tyr^47^, Pro^50^, Pro^90^, Leu^92^) of ACOT8, which are replaced by non-hydrophobic residues in *E. coli* isoenzyme (Glu, Gly, Ser and Lys respectively). The residues are coloured according to their physico-chemical properties (HKR in cyan, DE in red, STNQ in maroon, AVLIM in pink, FYW in blue, PG in orange and C in green). **(b)** The modelled ACOT8 dimer (chain A in blue and chain B in brownish). The homology model of ACOT8 was built based on the X-ray structure of the *E. coli* thioesterase. The two enzymes share about 41% of sequence identity.

For the modelling procedure, the pairwise sequence alignment between ACOT8 and the *E. coli* thioesterase (Fig. 1a) was extracted from the Hidden Markov profiles generated using the HHpred program^38^. A bundle of models comprising 100 decoys was generated and filtered using the Modeller scoring function. All the obtained three-dimensional models of ACOT8 did not deviate from currently available experimental geometries, indicating that both the secondary structures elements (12-stranded antiparallel β-sheets) and the typical tertiary fold (the double hot dog)^29^ were conserved in the model. The monomer-monomer interface between the two subunits of the dimer was totally buried in the protein, involving the central fragments of the six central β-sheets from the two monomers to form several stabilizing interactions, all conserved in the template (Fig. 1b). The contact surface was structurally similar to that of *E. coli* enzyme, i.e. 1980 and 2142 Å^2^ per monomer for the target and the template, respectively.

### Initial model of Nef/ACOT8 interaction

**Figure 2:**
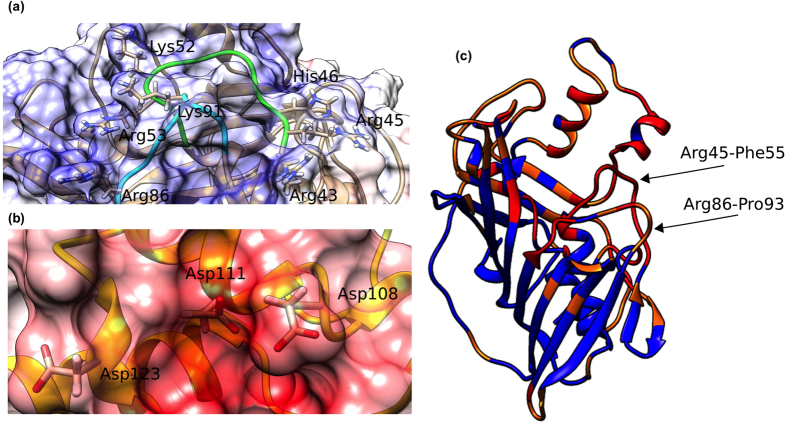
Electrostatic potentials and CPORT prediction. Electrostatic potentials on the ACOT8 **(a)** and the Nef **(b)** surfaces. The Nef/ACOT8 electrostatic complementarity can be vividly visualized by the plots of the electrostatic potential, calculated solving the Poison-Boltzmann equation^51^. The contact surfaces of Nef and ACOT8 are clearly complementary: while the surface of Nef is highly negative (red), the contact surface of ACOT8 is highly positively charged (blue). **(c)** CPORT prediction: the ACOT8 residues with a high prediction score are likely supposed to be involved in the interaction with Nef. In the figure, red/orange colour means a high prediction score, whereas blue colour means a low prediction score.

**Figure 3:**
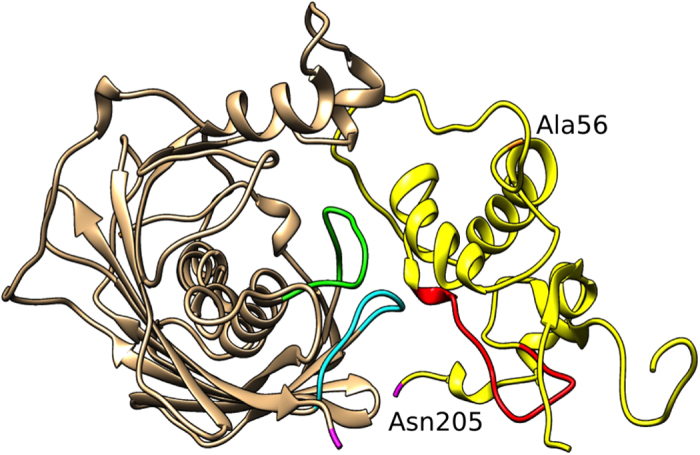
Docking model. Nef (yellow) and ACOT8 (brownish) docking model. The ACOT8 Arg^45^-Phe^55^ and Arg^86^-Pro^93^ regions are highlighted in green and in cyan respectively. For both proteins, N- and C-terminals are coloured in orange and magenta respectively. Nef amino acids, starting from Asp^123^, involved in proteasome degradation are highlighted in red.

The electrostatic potential on Nef/ACOT8 surfaces (Fig. 2a,b) showed a high electrostatic complementarity, i.e. a negatively charged surface on Nef-LAI and a positively charged one on ACOT8. In particular, the latter corresponded to one of the ACOT8 high probability interaction regions identified on the molecule through the CPORT (Consensus Prediction of interface Residues in Transient complexes) web server tool, which calculates a prediction score for each residue of the query protein, in this case ACOT8 (Fig. 2c). The residues with a high prediction score are supposed to be involved in the interaction with Nef. These computational predictions on ACOT8 and the available experimental data on Nef^30^,^31^, were combined together to guide the docking procedure using HADDOCK, to gain a better insight into the Nef/ACOT8 interaction (Fig. 3). HADDOCK results pointed principally to a most populated cluster (111/200 decoys) of structures that contained the best scoring models. Specifically, the root-mean-square-deviation (RMSD) of the backbone atoms from the overall lowest-energy structure was 0.64 ± 0.40 Å. A total of five clusters were obtained, with the second most populated comprising just 53/200 decoys, and while the HADDOCK score was about −222.6 for the first cluster, in the second it was −96.7. Thus, the representative structure of the most populated cluster was analysed in order to extract information on the residues present in the Nef/ACOT8 interface. In this cluster, salt bridges were found between Lys^52^ of ACOT8 and Asp^108^ and Asp^111^ of Nef-LAI, but also between Lys^91^ of ACOT8 and Asp^123^ of Nef. In addition to this salt bridge networks, 11 hydrogen-bond interactions occurred between Nef and ACOT8 and hydrophobic contacts were detected between three residues of Nef, i.e. Leu^112^, Phe^121^ and Pro^122^ and three residues of ACOT8, i.e. Tyr^47^, Pro^50^ and Pro^90^.

### Development of ACOT8 deletion mutants and their expression

Taking into account the hypothetical contact points identified from the Nef/ACOT8 complex model, two ACOT8 point mutants were produced in correspondence of Lys^52^ (p∆PAK) and Lys^91^ (p∆PK). A non-peroxisomal ACOT8 mutant was also obtained by point mutation of the peroxisomal target signal, from SKL^317–319^ to SKR^317–319^. To extend the comprehension of the Nef/ACOT8 association, the ∆PAK (Arg^45^-Phe^55^), ∆PK (Arg^86^-Pro^93^) and ∆PAK-∆PK (both Arg^45^-Phe^55^ and Arg^86^-Pro^93^) mutants were prepared, too.

### Coimmunoprecipitation assay between Nef and ACOT8 mutants

**Figure 4:**
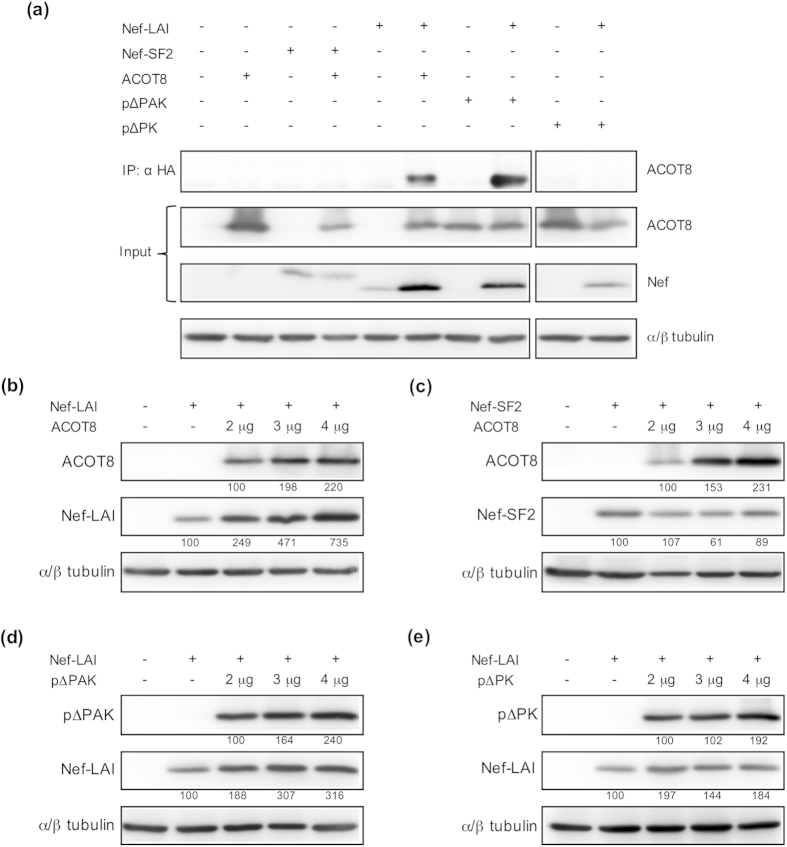
Coimmunoprecipitation assay between Nef and ACOT8 mutants. **(a)** CHO cells were cotransfected with Nef-LAI and ACOT8 mutants as indicated in the panel. Nef-SF2 was used as negative control. Whole cell extracts were quantified and analysed with anti-HA or anti-ACOT8 antibodies, as indicated. α/β tubulin expression was used as a control. The Nef/ACOT8 mutants association was analysed by immunoprecipitation of the complexes with the anti-HA antibody. Western blot analysis of immunoprecipitated lysates, performed with the anti-ACOT8 antibody, shows that only the wild type ACOT8 and the p∆PAK mutant interact with Nef-LAI. CHO cells were cotransfected with either Nef-LAI **(b)** or Nef-SF2 **(c)** and increasing amounts of ACOT8. Similarly, they were cotransfected with increasing amount of either the p∆PAK **(d)** or the p∆PK mutant **(e)** and Nef-LAI. ACOT8 and Nef expression was analysed by Western blot. α/β tubulin expression was used as control. Nef and ACOT8 expression was calculated from the same membrane by densitometry analysis using the ImageJ software and normalized to α/β tubulin. Percentages relative either to the minimum amount of transfected ACOT8 construct, or to the Nef transfection, are indicated under each western blot image.

To assess the association between Nef and the ACOT8 point mutants, a coimmunoprecipitation assay was performed. CHO cells were cotransfected with plasmids encoding for ACOT8 mutants (p∆PAK and p∆PK) and HA-tagged Nef. The complexes were immunoprecipitated with an anti-HA antibody. Western blot analysis of the immunoprecipitated lysates (Fig. 4a) confirmed that no interaction occurred between Nef from HIV-1 SF2 strain (Nef-SF2) and ACOT8, while a strong association was detected after coexpression of Nef-LAI with ACOT8, confirming previous data^30^. Analysing the association between Nef-LAI and ACOT8 mutants, we observed that the pΔPAK mutant was present in immunocomplexes with Nef-LAI, suggesting that the ACOT8 Lys^52^ may not be involved in the interaction. On the contrary, the pΔPK mutant was not found associated with Nef-LAI, indicating that the ACOT8 Lys^91^ may play a key role in the complex formation. Western blot analysis of the whole protein extracts (input) showed that Nef-LAI expression was increased in the presence of ACOT8, as well as the p∆PAK mutant. This effect was not observed either for Nef-SF2 or for the non-interacting p∆PK mutant.

### Analysis of the influence of ACOT8 on Nef expression

To investigate the effect of ACOT8 on Nef expression levels, CHO cells were cotransfected with Nef-LAI and increasing amounts of ACOT8 (Fig. 4b). Western blot analysis indicated that the amount of Nef-LAI detected was directly correlated to ACOT8 expression: the more ACOT8 is expressed, the more Nef-LAI is detected. On the contrary, when CHO cell were cotransfected with the non interacting Nef-SF2 and increasing amounts of ACOT8 (Fig. 4c), Nef-SF2 is similarly express. When Nef-LAI was cotransfected with increasing amounts of either p∆PAK or p∆PK ACOT8 mutants, the Nef-LAI expression levels were related to the p∆PAK expression (Fig. 4d) while, in the presence of the p∆PK mutant (Fig. 4e), unable to interact with Nef-LAI, such correlation was not observed.

### Nef and ACOT8 mutants colocalization

**Figure 5:**
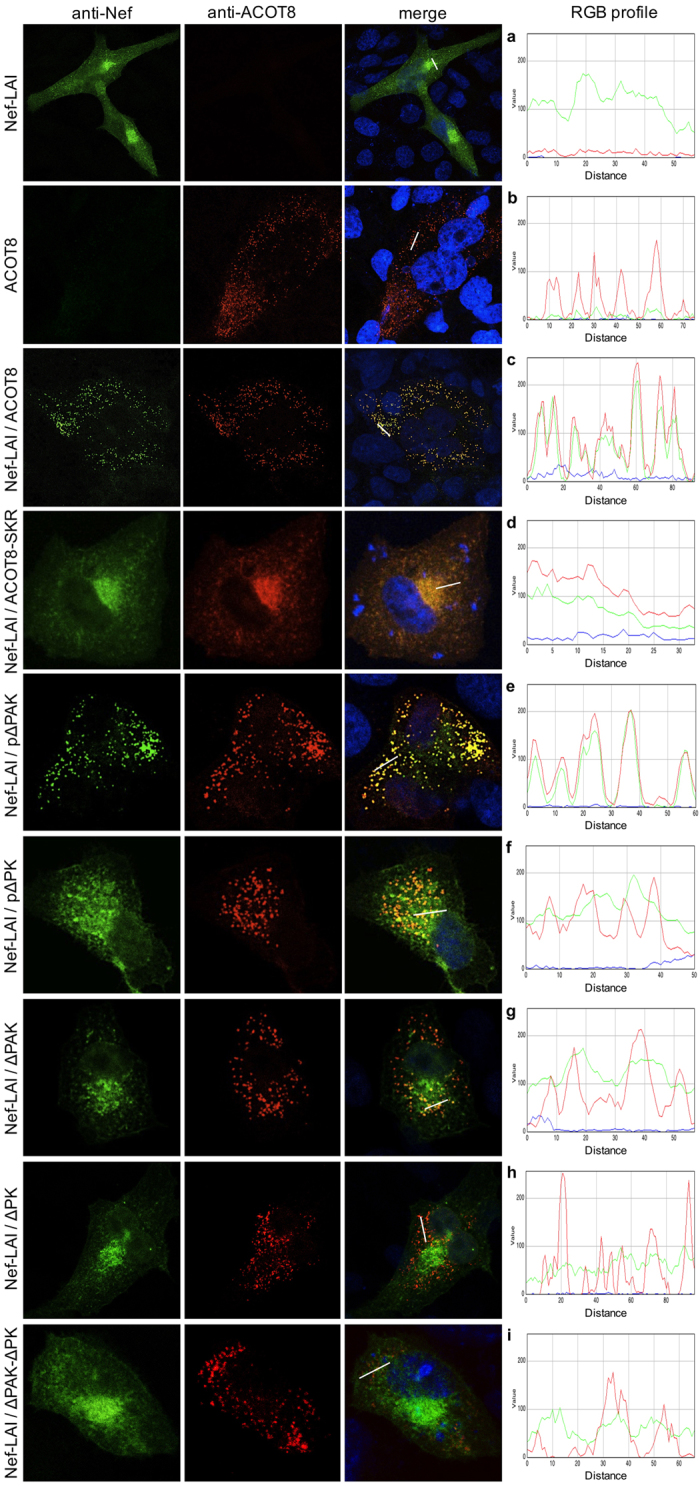
Colocalization assay between Nef and ACOT8 mutants. CHO cells cotransfected with Nef-LAI **(a)**, ACOT8 **(b)**, Nef-LAI and ACOT8 **(c)**, Nef-LAI and ACOT8-SKR **(d)**, Nef-LAI and p∆PAK **(e)**, Nef-LAI and p∆PK **(f)**, Nef-LAI and ∆PAK **(g)**, Nef-LAI and ∆PK **(h)**, Nef-LAI and ∆PAK-∆PK **(i)**. Nef was detected using an anti-HA antibody (green signal); ACOT8 was detected using an anti-ACOT8 antibody (red signal). Merge images represent the images of the localization of Nef and ACOT8 overlaid. Overlap of Nef and ACOT8 results in a yellow colour. On the right side, the RGB profile plotted along the line drawn in the merged image is shown. Merge and single-channel images come from a single z-plane.

**Figure 6:**
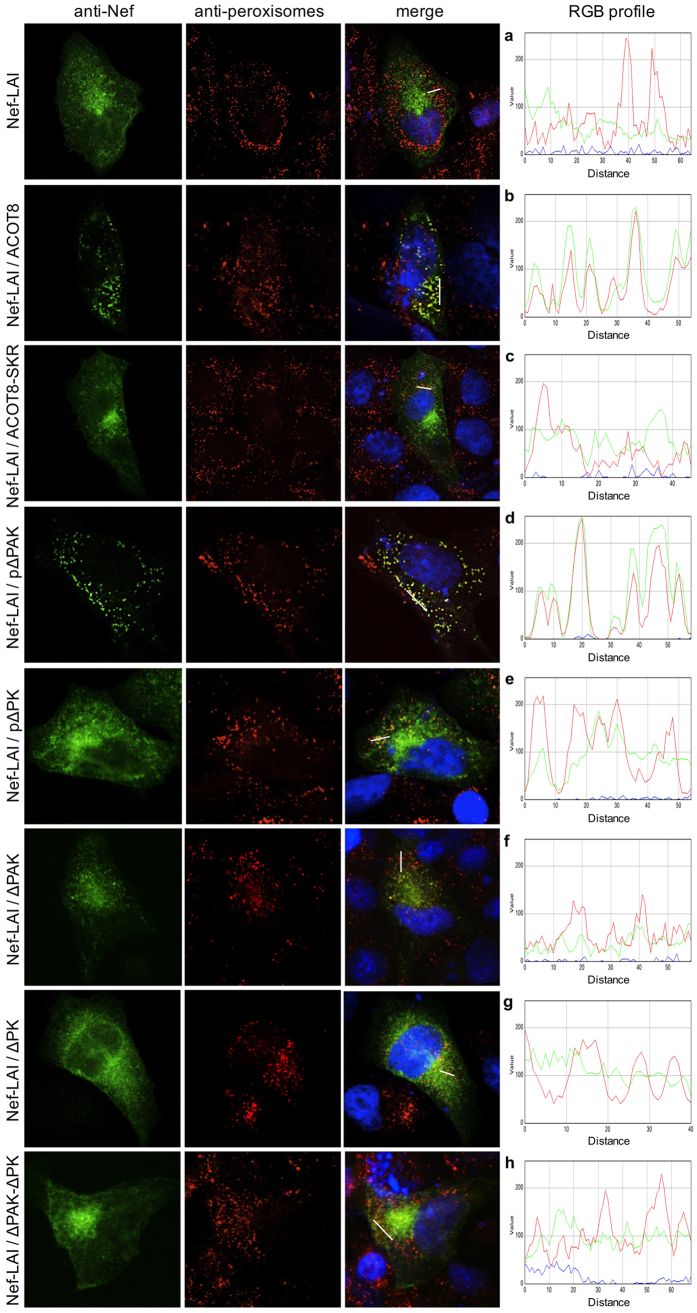
Nef localization in peroxisomes in the presence of ACOT8 mutants. CHO cells transfected with Nef-LAI **(a)**, Nef-LAI and ACOT8 **(b)**, Nef-LAI and ACOT8-SKR **(c)**, Nef-LAI and p∆PAK **(d)**, Nef-LAI and p∆PK **(e)**, Nef-LAI and ∆PAK **(f)**, Nef-LAI and ∆PK **(g)**, Nef-LAI and ∆PAK-∆PK **(h)**. Nef was detected using an anti-HA antibody (green signal); peroxisomes were detected using an anti-peroxisomes antibody (red signal). Merge images represent the images of the localization of Nef and peroxisomes overlaid. Overlap of Nef and peroxisomes results in a yellow colour. On the right side, the RGB profile plotted along the line drawn in the merged image is shown. Merge and single-channel images come from a single z-plane.

To better explore the interaction between Nef and ACOT8, CHO cells were cotransfected with Nef-LAI and ACOT8 mutants. Through immunofluorescence it was assessed their association (Fig. 5), as well as the Nef localization in peroxisomes (Fig. 6). Immunostaining of Nef-LAI revealed a cytoplasmic distribution, with a prevalent localization at the perinuclear region (Fig. 5a), while ACOT8 showed a clear peroxisomal localization (Fig. 5b). When the two proteins were coexpressed, Nef-LAI was completely targeted to peroxisomal regions, where the association with ACOT8 occurs (Fig. 5c). In the presence of the non-peroxisomal ACOT8-SKR mutant, the association with Nef-LAI was maintained, but it was not localized in peroxisomes (Fig. 5d).

Nef-LAI and pΔPAK colocalization signal did not differ from that observed in the Nef-LAI/ACOT8 cotransfected cells (Fig. 5e). On the contrary, the Nef-LAI association was partially lost with the pΔPK mutant (Fig. 5f).

The ΔPAK mutation resulted in a loss of association with Nef-LAI (Fig. 5g), which was abrogated in the presence of the ΔPK mutant (Fig. 5h); as a consequence, Nef-LAI remained prevalently localized at the perinuclear region. The double ΔPAK-ΔPK mutation completely abolished Nef/ACOT8 interaction (Fig. 5i).

Peroxisomal staining indicated that Nef-LAI was not localized in the peroxisomes (Fig. 6a), but when cotransfected with ACOT8 it was relocated in the peroxisomes (Fig. 6b). The non-peroxisomal mutant (ACOT8-SKR) lost the ability to target Nef-LAI in these organelles (Fig. 6c). Transfection of the pΔPAK mutant still induced the Nef-LAI peroxisomal localization (Fig. 6d), while in the presence of the pΔPK mutant no labelling of Nef-LAI in the peroxisomes was detected (Fig. 6e). The ΔPAK mutation resulted in a reduced Nef-LAI peroxisomal targeting (Fig. 6f), which appeared to be more severe in the presence of the ΔPK mutant (Fig. 6g). The ΔPAK-ΔPK mutant completely abrogated the colocalization between Nef-LAI and peroxisomes (Fig. 6h). Overall, the results obtained analysing both the Nef/ACOT8 and the Nef/peroxisomes colocalization were comparable.

### Comparison between Nef-LAI and Nef-SF2

**Figure 7:**
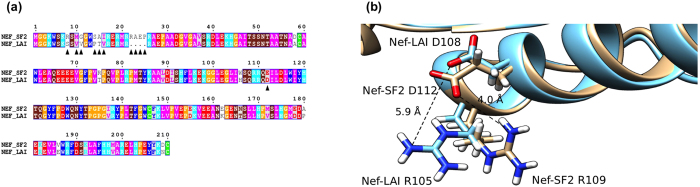
Sequence alignment between Nef-SF2 and Nef-LAI. **(a)** ▲ indicates some residues differences between the two alleles in the N-terminal region and in Glu^112^ corresponding to Asp^108^ in Nef-LAI. The residues are coloured according to their physico-chemical properties (HKR in cyan, DE in red, STNQ in maroon, AVLIM in pink, FYW in blue, PG in orange and C in green). **(b)** Superimposition of Nef-LAI structure (in cyan) and Nef-SF2 structure (in brownish) shows that, while Nef-SF2 Glu^112^ might interact with the Nef-SF2 Arg^109^ due its longer side-chain, Nef-LAI Asp^108^ (shorter side-chain) could not be involved in an intramolecular salt bridge with Nef-LAI Arg^105^, remaining free to interact with ACOT8.

Nef-LAI and Nef-SF2 differ by 30 aminoacidic residues. Analysing the sequence alignment (Fig. 7a), and in particular the 108–124 Nef-LAI region, it was observed that Asp^108^ is swapped in glutamic acid in SF2 (residue 112 in SF2 homologous). As reported by Cohen and collaborators, this mutation is responsible for the lack of interaction with ACOT8^30^. From the structural point of view, in Nef-SF2 the longer side-chain of the glutamic acid might form an intramolecular salt bridge interaction with the closest Arg^109^ (following the Nef-SF2 numbering system) (Fig. 7b). Consequently, the residue would not be available to interact with ACOT8, weakening a putative salt-bridge network.

It has been also proved that the N-terminal region of Nef-LAI could be involved in the interaction or in the stabilization of the Nef/ACOT8 complex^30^. Unfortunately, this region was not solved for the Nef-LAI crystal structure, and therefore it was not possible to include it in the model.

### Knowledge-based Nef/ACOT8 complex model

**Figure 8:**
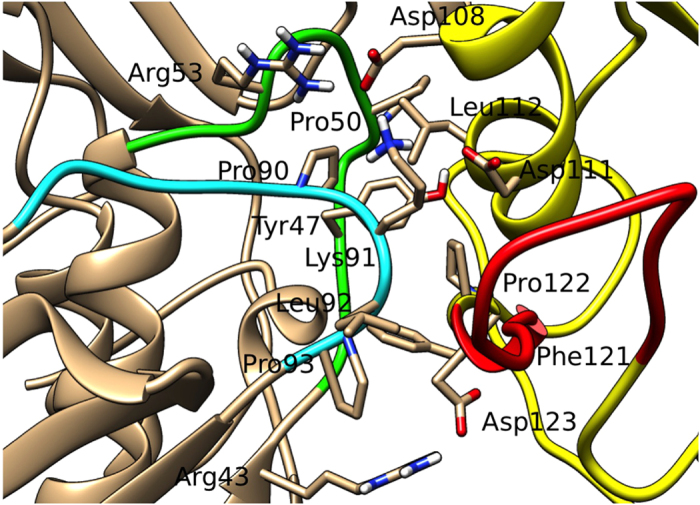
Detail of the knowledge guided Nef/ACOT8 complex. Residues involved in the Nef/ACOT8 interaction, characterized in this work for ACOT8 and by Liu^31^ and Cohen^30^ for Nef, are shown. The colour code is the same as in Fig. 3.

To model the Nef/ACOT8 complex, in order to explain all the available experimental data, a new virtual protein-protein docking between the structural model of Nef-LAI and ACOT8 was carried out. The docking procedure was guided with our *in vitro* experimental data, resulting in three different clusters of decoys. The best score model of the most populated cluster (165/200 decoys) was analysed. In particular, several pairs of amino acids were identified showing a high electrostatic complementarity. Indeed, again, while ACOT8 shows a positively charged interaction surface, formed principally by Arg^43^, Arg^45^, His^46^, Lys^52^, Arg^53^, Arg^86^ and Lys^91^, Nef negatively charged surface is formed by Asp^108^, Asp^111^, Asp^123^. In particular, salt bridges were found between ACOT8 Lys^91^ and Nef Asp^108^ and Asp^111^, and also between ACOT8 Arg^53^ and Nef Asp^108^, and finally between the ACOT8 Arg^43^ and Nef Asp^123^. In this Nef/ACOT8 model, while residues in the Arg^45^-Phe^55^ loop (deleted in the ∆PAK mutant) seem to be involved in complex stabilization, Lys^52^ is not involved in the interaction, as observed by *in vitro* experiments. Several intermolecular hydrophobic contacts were also formed between three residues of Nef, i.e. Leu^112^, Phe^121^ and Pro^122^ and five residues of ACOT8 i.e. Tyr^47^, Pro^50^, Pro^90^, Leu^92^ and Pro^93^, allowing a further stabilization of the complex (Fig. 8).

This hypothesis could be supported by the observation that some of these hydrophobic amino acids, i.e. Tyr^47^, Pro^50^, Pro^90^, Leu^92^, are lacking in the *E. coli* thioesterase and this may be the reason why the interaction with Nef does not take place, as previously observed by Cohen and colleagues^30^. In fact, in the *E. coli* thioesterase, these residues are substituted by non-hydrophobic residues (Glu, Gly, Ser and Lys respectively), completely changing the physico-chemical properties of the interaction surface (Fig. 1).

## Discussion

The association between HIV-1 Nef and the peroxisomal human thioesterase 8 (ACOT8) has been described^24^,^25^ and the Nef regions responsible for the association with ACOT8 have been characterized^30^,^31^. Current literature indicates that HIV-1 Nef is the only known viral protein interacting with ACOT8, but the structural properties of the ACOT8 regions interacting with Nef have never been characterized. Thus, for many years the role of the Nef/ACOT8 association remained unclear. Previous data reported that the interaction between Nef and ACOT8 results in a relocalization of Nef from its cytoplasmic distribution to the peroxisomes^30^. Recently Gondim *et al.*^39^ demonstrated that among the different known Nef interacting proteins, ACOT8 shows the strongest association. In the same study, it was reported that ACOT8 is not involved in CD4 downregulation, as previously hypothesized^25^,^32^. The study also showed that the Nef membrane localization is not strictly required for the interaction with ACOT8, suggesting that their association might occur early during HIV-1 infection. In addition, the authors observe that the Δ17–26 Nef mutant, which is known to be unable to downregulate MHC-I molecules^40^, does not interact with ACOT8, suggesting that ACOT8 might be involved in Nef-mediated MHC-I downregulation.

Our study aims to identify, for the first time, the critical ACOT8 residues involved in the interaction with Nef, an important step toward the elucidation of the biological role of their interaction. Bioinformatic tools allowed to predict the ACOT8 regions most likely involved in the interaction with Nef and to plan the construction of several mutants. It was then possible to generate a model able to explain all the experimentally identified residues involved in Nef/ACOT8 interaction^30^,^31^. Two point mutants were developed by deletion of Lys^52^ (pΔPAK mutant) and Lys^91^ (pΔPK mutant). A non-peroxisomal ACOT8 mutant was obtained by mutation of the peroxisomal target signal (PTS) 1, from SKL to SKR. Coimmunoprecipitation analysis confirmed that ACOT8 associated with Nef-LAI, but not with the Nef variant from HIV-1-SF2^30^. The pΔPAK mutant showed interaction with Nef-LAI similarly to wild type ACOT8, while the pΔPK mutant did not show any association. These findings indicate that the ACOT8 Lys^52^ (pΔPAK) is not fundamental for the binding, but the Lys^91^ may play a role in the Nef/ACOT8 association. In particular, western blot analysis indicated that the Nef-LAI detection was strictly correlated to ACOT8: the more ACOT8 is expressed, the more Nef-LAI is detected. On the contrary, no effect was observed when ACOT8 was cotransfected with Nef-SF2, suggesting that the ACOT8 association to Nef could have a stabilizing role for the Nef expression. This finding was further confirmed when Nef-LAI was cotransfected with ACOT8 mutants: increasing amount of the p∆PAK correlated with increasing expression of Nef-LAI, while no evident correlation was observed in the presence of the p∆PK mutant, which resulted unable to interact with Nef-LAI. This data, beyond confirming that Lys^91^ is important for interacting with Nef, point out that the Nef/ACOT8 association might have a functional role during HIV-1 infection, preventing the Nef degradation. Interestingly, it has been reported that, in the first step of HIV-1 replication, proteasome can interact with Nef^41^. Indeed, it has been demonstrated that the Nef 123 to 152 region (Nef^123–152^) is one of the immunogenic domains involved in proteasomal digestion and that the region comprising amino acids 128 to 135 is one of the principal cleavage products^42^. The Nef^123–152^ segment is highly conserved among different HIV-1 Nef variants^43^ (as indicated in Fig. 7a) but, since this region overlaps with the Nef/ACOT8 interaction surface, we hypothesize that when Nef binds ACOT8, the proteasome could not interact, probably due to steric hindrance.

To better explore the Nef/ACOT8 interaction, ACOT8 mutants harbouring wider deletions, either in the Arg^45^-Phe^55^ region (ΔPAK mutant), or in the Arg^86^-Pro^93^ region (ΔPK mutant), were prepared. A double mutant harbouring both ΔPAK and ΔPK deletions was also obtained (ΔPAK-ΔPK mutant). The interaction between ACOT8 mutants and Nef was further assessed by immunofluorescence. As expected, cotransfection of Nef-LAI and ACOT8 resulted in a strong association in the peroxisomes, according to previous data^30^. In the presence of the non-peroxisomal ACOT8 mutant, Nef-LAI remained associated with ACOT8-SKR, but their localization was widespread, highlighting the importance of the ACOT8 PTS1 sequence for the Nef peroxisomal localization. Analysing the effect of ACOT8 mutants, it was observed that in the presence of the pΔPAK mutant, the association with Nef-LAI was maintained and an ACOT8-like Nef peroxisomal localization was observed. The pΔPK mutant showed a reduced association with Nef-LAI and the Nef peroxisomal targeting was lost. The same effect was observed in the presence of the ΔPAK mutant. The ΔPK mutation abrogated the interaction with Nef-LAI, which showed a prevalent perinuclear localization. In the presence of the ΔPAK-ΔPK mutant the interaction with Nef-LAI was completely lost and no peroxisomal colocalization was detected.

Our data indicate that the ACOT8 Lys^91^ may play a key role, since its single mutation to Ser^91^ is sufficient to abrogate the association with Nef. In particular, the positive charge of Lys^91^ may have a fundamental role in maintaining the charge complementarity observed in the docking model, which appears to be crucial for the association with Nef.

However, the observation that both Arg^45^-Phe^55^ and Arg^86^-Pro^93^ may be determinant for Nef association suggests that other residues may contribute to the Nef binding, meaning that more than a single interaction occurs. This is in agreement with previous speculations indicating that a cooperative effect might subsist between separated points of interaction^30^.

All the experimental data were finally summarized in a Nef/ACOT8 model (Fig. 8), where the ACOT8 Lys^91^ resulted to interact with Asp^108^ and Asp^111^ of Nef-LAI, while the ACOT8 Lys^52^, as experimentally observed, did not appear to be involved in the interaction. Moreover, in this model, the ACOT8 Arg^53^ showed electrostatic interactions with Nef-LAI Asp^108^, validating the observation that the ∆PAK mutation (Arg^45^-Phe^55^) abrogated the interaction with Nef. Our predictions were further supported by the interesting observation that in Nef-SF2, not interacting with ACOT8, the residue corresponding to Nef-LAI Asp^108^, which resulted to be necessary for the formation of salt bridges with ACOT8 Lys^91^, is swapped in glutamic acid. Currently, the biological function of the association between Nef and ACOT8 has not yet been elucidated. Several hypotheses have been proposed, suggesting that ACOT8 may be involved in the control of lipid modifications of proteins, which are important for membrane anchoring of proteins and receptor internalization. In addition, through the intracellular regulation of acyl-CoA, ACOT8 may influence Nef myristoylation. The Nef/ACOT8 association may also ensure an efficient HIV-1 virions budding, modifying the composition of membrane lipids. Interestingly, it has been reported that Nef plays a significant role in pathogenesis of lipid-related metabolic complications of HIV-1 infection^44^. However, whether Nef has any need to be localized to peroxisomes is not clear. It has been hypothesized that when Nef is present in excess over ACOT8, such as in HIV-1-infected cells, the Nef/ACOT8 complex might colocalize predominantly outside the peroxisomes^30^.

Our data support the hypothesis that the Nef association to ACOT8 may improve the Nef stability, probably preventing its proteasome-mediated degradation. In conclusion, our results indicate that in the absence of an experimentally determined structure, as in the case of ACOT8, computational models, within their limitations, are often helpful for generating testable hypotheses and giving insight into existing experimental data^45^,^46^,^47^. This experimental/computational combined study, through the identification of the ACOT8 regions most likely involved in the interaction with Nef (Arg^45^-Phe^55^ and Lys^91^), elucidates the association between HIV-1 Nef and human ACOT8. Our data open the way for further studies aimed at improving the comprehension of the biological role of their interaction. ACOT8 mutants originated and characterized in this study, may help to elucidate the role of the interaction in ACOT8-knock out cell lines.

Since any ACOT8 deficiency has been correlated to lipid metabolism impairment, due to the human thioesterase redundancy, the development of ACOT8 inhibitors could be exploited as a novel strategy to interfere with several Nef cellular functions as well as with the HIV-1 infective life cycle.

## Methods

### ACOT8 homology modelling

To obtain the ACOT8 model, protein sequences of all members of the ACOT8 family were retrieved from the Uniprot database. A multiple sequence alignment was built using the PROMALS program^48^,^49^. The sequences were used for the definition of a Hidden Markov Model (HMM) profile of the ACOT8 family using the HHsearch program^49^. All alignments were displayed using the ESPript 3.0 web-server^50^. The multiple sequence alignment obtained was used as reference for the structural prediction of ACOT8 by homology modelling based on the crystal structure of the *E. coli* thioesterase [PDB: 1C8U] and the dimeric functional configuration was generated and relaxed using the Modeller9v3 program^46^.

### Prediction Nef/ACOT8 interface and Dockings: CPORT-HADDOCK

To model the Nef/ACOT8 interaction, the crystal structure of Nef-LAI [PDB: 1AVV] was used. Since the residues from 148–178 are missing, this region was integrated using the structure of Nef-LW123 [PDB: 2NEF]. In both structures the experimental binding region was conserved and the inserted loop was far from it. The Nef/ACOT8 electrostatic potentials were calculated using the APBS program^51^. The CPORT (Consensus Prediction Of interface Residues in Transient complex) bioinformatic tool, a consensus method combining six interface web servers (WHISHY, PIER, ProMate, cons-PPISP, SPIDER and PINUP), was used as interface prediction program^52^. The Nef/ACOT8 docking model was then performed using the HADDOCK (High Ambiguity Driven biomolecular DOCKing) version 2.1 program^53^,^54^, based on the previously described bioinformatics predictions. These latter were introduced as Ambiguous Interaction Restraints (AIRs) to drive the docking process. The docking was performed with default parameters using the web-server version of HADDOCK. All calculations were performed with CNS1.2^55^. Non-bonded interactions were calculated with the OPLS force field using a cut-off of 8.5 Å^56^. The electrostatic potential was calculated using a shift function, while a switching function (between 6.5 and 8.5 Å) was used to define the Van der Waals potential. The ambiguous interaction restraints to drive the docking were defined considering as Nef active residues Asp^108^, Leu^112^, Phe^121^, Pro^122^ and Asp^123 ^30,31, whereas for ACOT8 the active residues were Arg^43^, Arg^45^, His^46^, Lys^52^, Arg^53^, Arg^86^ and Lys^91^. They were chosen considering both the charge complementarity between Nef and ACOT8, and the CPORT prediction. Passive residues were defined automatically via the web server as those surrounding the active ones. Docking results were visualized using the CHIMERA program^57^.

### Knowledge-based Nef/ACOT8 model

To drive the second docking, the amino acids considered as active for Nef were the same as the first docking and no passive amino acids were defined. For ACOT8, only Lys^91^ was chosen as active residue and Arg^86^, Ala^87^, Gly^88^, Asp^89^, Pro^90^, Leu^92^ and Pro^93^ were defined as passive residues.

### Cell lines

CHO (Chinese Hamster Ovary) cells were obtained from the American Type Culture Collection (ATCC). They were grown at 37 °C in a humidified atmosphere with 5% CO_2_ and maintained in Dulbecco’s Modified Eagle’s Medium supplemented with 10% FBS, 2mM L-Glutamine, 100 U Penicillin/ml and 100 U Streptomycin/ml. Mycoplasma testing was routinely performed. CHO cells were chosen since endogenous ACOT8 differs from human ACOT8 in the N-terminal region, which is the epitope recognized by the anti-ACOT8 antibody used.

### Vectors

HIV-1-LAI Nef (Nef-LAI) and HIV-1-SF2 Nef (Nef-SF2) plasmids were provided by M. Pizzato (CIBIO, Trento, Italy). Both sequences harbour a C-terminal HA-tag^58^. The ACOT8 plasmid was provided by S. Benichou (Institut Cochin de Genetique Moleculaire, France)^24^.

### ACOT8 mutants

The p∆PAK mutant has a point deletion of Lys^52^; the p∆PK has a missense mutation from Lys^91^ to Ser^91^. The ∆PAK was obtained by deleting Arg^45^-Phe^55^, the ∆PK by deleting Arg^86^-Pro^93^ and the ∆PAK-∆PK double mutant by introducing both deletions. Each mutant was obtained from the wild type *ACOT8* gene by PCR amplification of the fragment upstream and downstream each deletion. Primer pairs were developed to join the two fragments with specific restriction sites. The p∆PAK and ∆PAK mutants harboured the *Kas*I restriction site both in the reverse primer of the upstream fragment (5′-*GGCGCC*TACCCAGTAATGCCTTCC-3′ and 5′-*GGCGCC*TCCTCTGAAGAGATCCTC-3′ respectively) and in the forward primer of the downstream fragment (5′-*GGCGCC*AGGCTGTTTGGTGGTCAG-3′ and 5′-*GGCGCC*GGTGGTCAGATCGTGGGC-3′ respectively). The p∆PK and ∆PK mutants harboured the *Bmt*I site both in the reverse primer of the upstream fragment (5′-*GCTAGC*GTCCCCTGCCCGAACAAA-3′ and 5′-*GCTAGC*AACAAAGTAGCAGTGCAG-3′, respectively) and in the forward primer of the downstream fragment (5′-*GCTAGC*CTGCCAGTACTGTACCAA-3′ and 5′-*GCTAGC*GTACTGTACCAAGTGGAG-3′, respectively). The ∆PAK-∆PK double mutant was obtained from the ∆PAK by PCR amplification of the fragments upstream and downstream the ∆PK deletion. To insert the full constructs in pcDNA3, the forward primer of the upstream fragments harboured the *Hind*III site (5′-*CTATAG*GGAGACCCAAGCTT-3′) and the reverse primer of the downstream fragments harboured the *Apa*I site (5′-TT*GGGCCC*TCTGGCTACAGCTTGC-3′). Each fragment pair was joined through *Kas*I or *Bmt*I sites and the full fragment was cloned in pcDNA3 through *Hind*III and *Apa*I sites. The ACOT8-SKR mutant, missing the peroxisomal localization signal, was developed by PCR inserting a SKL to SKR mutation. A reverse primer (5′-*GGGCCC*TCTGGCTAC*C*GCTTGC-3′) harbouring the mutation was used. The fragment was cloned in pcDNA3 through *Hind*III and *Apa*I restriction sites. Each construct was verified by direct sequencing.

### Antibodies

The anti-HA mouse antibody (Covance) was used to detect HA-tagged Nef, while the anti-ACOT8 N-19 goat antibody (Santa Cruz Biotechnologies) was used to detect the expression of ACOT8 mutants. Tubulin was detected using the anti α/β-tubulin antibody (Cell Signaling); the anti-PMP-70 antibody from guinea pig^59^ was used for peroxisomal staining. HRP-conjugated anti-mouse (Promega), HRP conjugated rabbit anti-sheep (Thermo Scientific) and HRP-conjugated goat anti-rabbit (PerkinElmer) antibodies were used for western blot assay. For immunofluorescence, the Alexa Fluor 488 conjugated goat anti-mouse (Cell Signaling), the Alexa Fluor 594 conjugated donkey anti-goat (Life Technologies) and the Alexa Fluor 555 conjugated goat anti-guinea pig (Life Technologies) antibodies were used.

### Transfections

Cells were transfected with the TransIT-LT1 transfection reagent (Mirus Bio), following manufacturer’s instructions.

### Coimmunoprecipitation assay

Cells (2.8 × 10^5^) were seeded on 60 mm dishes, cotransfected the following day with both 2.5 μg of either Nef-LAI or Nef-SF2 expressing plasmid and 4 μg of the ACOT8 constructs. Total DNA was compensated to the same amount with the pcDNA3 empty vector. Coimmunoprecipitation was performed 48 hours post-transfection as previously described^60^. Briefly, cells were harvested and washed in ice cold PBS 1X, resuspended in 240 μl of non-denaturing lysis buffer (10 mM Tris–HCl, pH 7.5, 5 mM EDTA, 150 mM NaCl, 1% Triton X-100) and incubated for 10 minutes on ice. Lysates were sonicated twice for 5 seconds and frozen at −20 °C for 1 hour. Cellular debris was removed by centrifugation. Proteins obtained were quantified and incubated overnight at 4 °C with 2 μg of anti-HA antibody, and immunocomplexes were linked to Dynabeads^®^ protein G (Life Technologies) at 4 °C for 30 minutes. Beads were washed with PBS 1X and resuspended in elution buffer containing NuPAGE loading buffer (Life Technologies) and 0.25 mM dithiothreitol for western blot analysis.

### Western blot analysis of Nef/ACOT8 coexpression

CHO cells (2.8 × 10^5^) were seeded on 60 mm dishes and cotransfected the following day with both 2.5 μg of either Nef-LAI or Nef-SF2 expressing plasmid and increasing amounts of the ACOT8 construct, as well as increasing amount of the p∆PAK and p∆PK constructs (2 μg, 3 μg, 4 μg). Total DNA was compensated to the same amount with the pcDNA3 empty vector. Forty-eight hours after transfection, cells were lysed in RIPA buffer and proteins were quantified. Equivalent amounts of proteins (15 μg) were treated with 3% β-mercaptoethanol and separated by SDS-PAGE on a 10% acrylamide gel. Nef and ACOT8 were detected using either the anti-ACOT8 antibody or the anti-HA antibody, followed by HRP-conjugated anti-mouse or anti-sheep antibody incubation, respectively. As a control, α/β-tubulin was used. The signal was developed using the ECL Advance^TM^ Western Blotting Detection Kit (Amersham), through the AutoChemi System UVP (BioImaging System). Densitometry analysis was performed using the ImageJ software. Relative Nef and ACOT8 band intensity was normalized to α/β-tubulin from the same membrane.

### Immunofluorescence assay

Cells (2 × 10^4^) were seeded on 8-well CultureSlide (BD Falcon), cotransfected the following day with both 250 ng of Nef-LAI expressing plasmid and 400 ng of each ACOT8 construct; 48 hours post-transfection, cells were fixed with 2% paraformaldehyde and permeabilized with 0.3% Triton X-100. Colocalization analysis of Nef-LAI with ACOT8 mutants was performed with the anti-HA antibody, followed by the Alexa Fluor 488 conjugated goat anti-mouse antibody, and with the N-19 antibody, followed by Alexa Fluor 594 conjugated donkey anti-goat antibody incubation. For peroxisomes labelling, cells were stained with the anti-PMP-70 antibody, followed by Alexa Fluor 555 conjugated goat anti-guinea pig antibody. Control samples, in which the primary antibody for Nef, ACOT8 or PMP-70 was left out, were included in each experiment to exclude any antibody cross-reactivity. Nuclei were labelled with 1 μg/ml Hoechst in PBS. Images were captured using a confocal laser-scanning fluorescence microscope Leica TCS SP5 with a HCX PL APO objective, 1.4 numerical aperture, 63 × magnification and analysed with LAS AF software (Leica). RGB profile was calculated using the ImageJ software, with the RGB profiler plugin.

## Additional Information

**How to cite this article**: Serena, M. *et al.* Molecular characterization of HIV-1 Nef and ACOT8 interaction: insights from *in silico* structural predictions and *in vitro* functional assays. *Sci. Rep.* **6**, 22319; doi: 10.1038/srep22319 (2016).
